# Physical function and mental health trajectories in COVID-19 patients following invasive mechanical ventilation: a prospective observational study

**DOI:** 10.1038/s41598-023-41684-3

**Published:** 2023-09-04

**Authors:** Hiromasa Yamamoto, Shinya Tanaka, Daisuke Kasugai, Miho Shimizu, Yohei Tsuchikawa, Yuto Hori, Yuki Fugane, Takayuki Inoue, Motoki Nagaya, Norihito Omote, Michiko Higashi, Takanori Yamamoto, Naruhiro Jingushi, Atsushi Numaguchi, Yukari Goto, Yoshihiro Nishida

**Affiliations:** 1 Department of Rehabilitation, Nagoya University Hospital, Nagoya, Japan; 2 Department of Emergency and Critical Care Medicine, Nagoya University Graduate School of Medicine, Tsurumai-Cho 65, Syowa-Ku, Nagoya, Japan; 3 Department of Rehabilitation, Mie University Hospital, Tsu, Japan; 4 Department of Respiratory Medicine, Nagoya University Graduate School of Medicine, Nagoya, Japan; 5 Department of Orthopaedic Surgery, Nagoya University Graduate School of Medicine, Nagoya, Japan

**Keywords:** Infectious diseases, Quality of life, Respiratory signs and symptoms, Outcomes research

## Abstract

This prospective observational cohort study was performed to investigate the physical function and mental health trajectories of novel coronavirus disease 2019 (COVID-19) patients requiring invasive mechanical ventilation (IMV) after discharge from the intensive care unit (ICU). The study population consisted of 64 patients (median age, 60 years; 85.9% male; median IMV duration, 9 days). At ICU discharge, 28.1% of the patients had Medical Research Council (MRC) sum score < 48 points, and prolonged IMV was significantly associated with lower MRC sum score and handgrip strength. Symptoms were similar between groups at ICU discharge, and the symptoms most commonly reported as moderate-to-severe were impaired well-being (52%), anxiety (43%), tiredness (41%), and depression (35%). Although muscle strength and mobility status were significantly improved after ICU discharge, Edmonton Symptom Assessment System score did not improve significantly in the prolonged IMV group. EuroQol five-dimension five-level summary index was significantly lower in the prolonged than short IMV group at 6 months after ICU discharge. We found substantial negative physical function and mental health consequences in the majority of surviving COVID-19 patients requiring IMV, with prolonged period of IMV showing greater negative effects not only immediately but also at 6 months after discharge from the ICU.

## Introduction

The novel coronavirus disease 2019 (COVID-19) pandemic caused by severe acute respiratory syndrome coronavirus 2 (SARS-CoV-2) has placed severe burdens on the capacities of healthcare systems worldwide, with 567 million confirmed cases and more than 6 million deaths^1^. The clinical spectrum of COVID-19 ranges from mild to severe, and one meta-analysis reported an estimated in-hospital mortality rate of approximately 45% for cases of severe COVID-19 requiring invasive mechanical ventilation (IMV)^2^. Patients with severe COVID-19 who survive often require prolonged IMV support in the intensive care unit (ICU)^3^.

Prolonged IMV in known to have adverse effects on both physical and mental health in survivors of acute respiratory failure^4,5^, who can develop post-intensive care syndrome characterized by long-term physical, psychological, and cognitive sequelae that can persist for months to years^4^. Patients with COVID-19 requiring prolonged IMV have been reported to have longer stays in the ICU and to require deep sedation, neuromuscular blockade, and/or placement in the prone position, all of which are significant risk factors for ICU-acquired weakness^6^, and many of these patients develop impairments in physical function and limited mobility after ICU or hospital discharge^7,8^. In addition, severe COVID-19 have been reported to be associated with adverse mental health impacts, including severe anxiety and depression^9^, the severities of which were reported to be strongly correlated^10^. As these factors are related to both prognosis and subsequent quality of life (QOL), detailed assessment is necessary to facilitate appropriate treatment of surviving COVID-19 patients with respiratory failure requiring IMV. However, both physical function and mental health are usually assessed based only on a single measurement or on changes occurring only in hospital or after discharge, and the association between prolonged IMV and physical and mental health trajectories after discharge from the ICU have yet to be elucidated.

This study was performed to investigate the physical and mental health trajectories of COVID-19 patients following prolonged IMV after discharge from the ICU and from hospital.

## Methods

### Study population

All consecutive patients admitted to the ICU of Nagoya University Hospital between March and September 2021 due to COVID-19 with length of stay (LOS) > 24 h in the ICU were evaluated for inclusion in this single-center, prospective, observational study according to the following eligibility criteria: age ≥ 18 years; positive for COVID-19 with respiratory failure requiring IMV. The details of our clinical setting and management of COVID-19 were reported previously^11^. The diagnosis of COVID-19 was confirmed by real-time polymerase chain reaction for SARS-CoV-2 from any specimen. Management strategies for COVID-19 requiring IMV in the ICU were centered on the “ABCDEF” bundle^12^, and patients requiring < 4 L of oxygen were transferred to the COVID-19 general ward. The exclusion criteria were patients who died in the ICU, those who were not intubated, and those who did not receive rehabilitation therapy in the ICU. The first stage of the rehabilitation therapy program performed by a multidisciplinary team critical care consisting of eight physical therapists wearing personal protective equipment consisted of positioning or range of motion exercises in patients with a Richmond agitation sedation scale score ≤  − 2. Patients whose condition stabilized proceeded to the second stage, which consisted of sitting on the edge of the bed, standing, transferring to a chair, and active muscle training until discharge from the ICU. A multidomain rehabilitation intervention was then applied on ICU discharge, which consisted of supervised rehabilitation therapy to improve strength, balance, mobility, and endurance. Patients who were transferred to the hospital immediately after discharge from the ICU did not undergo rehabilitation and functional assessments at hospital discharge on the COVID-19 general ward.

### Data collection

The clinical details of the patients at presentation and their demographic information and biochemical data at ICU admission were obtained from electronic medical records, and details of management in the ICU were extracted from the ICU patient information system (Fortec ACSYS; Phillips, Tokyo, Japan). The 4C (Coronavirus Clinical Characterisation Consortium) Mortality Score, which uses eight variables—age, sex, number of comorbidities, respiratory rate, peripheral oxygen saturation on room air, Glasgow Coma Scale, blood urea nitrogen, and C-reactive protein—was calculated for each patient at the time of admission to the ICU, as described previously^13^. In addition, the worst Acute Physiology and Chronic Health Evaluation II (APACHE II) and Sequential Organ Failure Assessment (SOFA) scores, which were also calculated within 24 h after admission to the ICU, were used in the analyses. The clinical frailty scale, with scores ranging from 1 (very fit) to 9 (terminally ill), was used to assess the degree of frailty prior to ICU admission^14^.

### Physical function

The physical function of each patient was evaluated at discharge from both the ICU and from hospital. Muscle strength was determined using on the Medical Research Council (MRC) sum score, which assesses the strength of each muscle group in the upper and lower limbs with scores for each muscle group ranging from 0 to 5 and higher scores indicating greater muscle strength (total score range: 0 = worst to 60 = best, minimal clinically important difference of 4 points)^6,15^. In addition, muscle strength was also assessed by measurement of handgrip strength with the patient performing two maximal isometric voluntary contractions of the hands for 3 s each for both hands with the elbow joint angle fixed at 90° flexion in the supine position using a Jamar dynamometer set to the second handle position (DHD-1 Digital Hand Dynamometer; Saehan Corporation, Seoul, South Korea). The analyses were performed using the greatest strength expressed as absolute value (in kg). Muscle weakness was defined as MRC sum score < 48 points, or handgrip strength < 11 kg for men and < 7 kg for women, respectively^16^. In addition, low handgrip strength was defined as handgrip strength < 28 kg for men and < 18 kg for women^17^. The grip and release test (GRT) and the foot tapping test (FTT), in which we measured the number of times the patient could flex and stretch their fingers in 10 s for each hand and tap the sole of the foot in 10 s for each foot while keeping the heel in contact with the floor and with the knees at 90° flexion, were performed with the patient in the supine position to evaluate upper and lower peripheral extremity motor function, respectively^18,19^. The highest scores obtained for both GRT and FTT were used in the analyses.

### Symptom burden

The self-administered Edmonton Symptom Assessment System (ESAS) questionnaire, a validated and reliable patient-reported outcome measures tool assessing the severity of nine common symptoms (anxiety, depression, drowsiness, lack of appetite, nausea, pain, shortness of breath, tiredness, and impaired well-being), was used to assess each patient’s symptoms at discharge from the ICU and from hospital. The patients rated each symptom on an 11-point numeric scale with scores ranging from 0 (absence of symptom) to 10 (worst possible symptom)^20^. The ESAS scores were classified according to severity as follows: 0, no symptoms; 1–3, mild; 4–6, moderate; and 7–10, severe^21^.

### Clinical outcomes

Clinical outcomes, including LOS in the ICU, unplanned ICU readmission, and the location of hospital discharge were included in the analysis. The ICU mobility scale score, an 11-point ordinal scale ranging from 0 (lying/passive exercises in bed) to 10 (independent ambulation), was calculated at the time of discharge from the ICU and hospital. The number of days taken to first mobilization (defined as ICU mobility scale score ≥ 3, i.e., sitting on the edge of the bed or higher) was assessed^22^. The Barthel Index routinely recorded in the nursing and rehabilitation summaries was used as a measure of activities of daily living (ADL) at the time of hospital discharge.

All patients were followed up prospectively at approximately 6 months after ICU discharge by mail-based surveys with the EuroQol five-dimension five-level (EQ-5D-5L) questionnaire and the EuroQol Visual Analogue Scale (EQ-VAS) as indices of QOL^23^. The EQ-5D-5L summary index was calculated based on responses related to five health dimensions (mobility, self-care, usual activities, pain/discomfort, and anxiety/depression), each of which was rated on a 5-point scale from 1 (best) to 5 (worst). The responses were translated using Japanese value sets into the EQ-5D-5L summary index, ranging from − 0.025 (worst) to 1 (best)^24^. EQ-VAS was used to record the patient’s self-rated health on a scale ranging from 0 (worst) to 100 (best).

### Statistical analysis

Continuous variables are expressed as the median and interquartile range (IQR), and categorical variables are expressed as numbers and percentages. As there was no established cutoff value for prolonged IMV in COVID-19 patients, the cohort was divided into the short IMV group and prolonged IMV group according to the median duration of IMV. Differences between groups were evaluated by the Mann–Whitney U test for continuous variables and Fisher’s exact test for dichotomous variables. Within-group differences in physical function and symptom burden between ICU discharge and hospital discharge were evaluated by Wilcoxon’s signed-rank test. The primary outcome was MRC sum score at ICU discharge.

Statistical analyses were performed using SPSS version 23.0 (IBM Corporation, Armonk, NY) and R version 3.2.1 (R Foundation for Statistical Computing, Vienna, Austria). In all analyses, a two-tailed *P* < 0.05 was taken to indicate statistical significance.

### Ethics approval and consent to participate

The study was approved by the Institutional Review Board of Nagoya University Hospital and was performed in accordance with the tenets of the Declaration of Helsinki and the Japanese Ethical Guidelines for Medical and Health Research Involving Human Subjects. Informed patient consent was obtained, and all participants were informed that they were free to opt out of participation in the study at any time.

## Results

**Figure 1:**
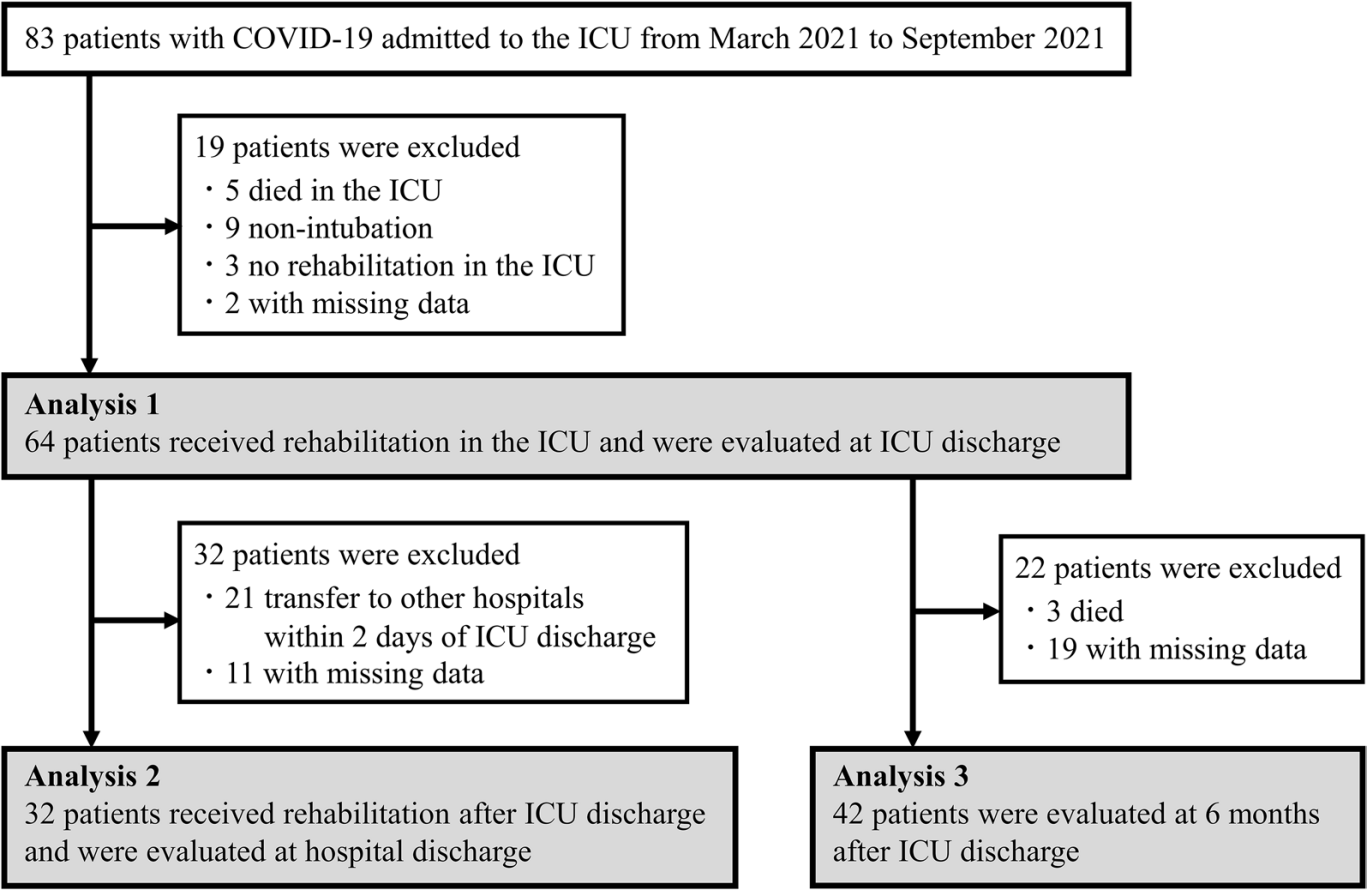
Flow chart of inclusion of patients in the study cohort. COVID-19—coronavirus disease 2019, ICU—intensive care unit.

A total of 83 consecutive critically ill patients with laboratory confirmed COVID-19 were admitted to the ICU of Nagoya University Hospital between March 2021 and September 2021. After excluding 19 patients (5 who died in the ICU, 9 without intubation, 3 who did not receive rehabilitation therapy in the ICU, and 2 with missing data), 64 patients requiring IMV were finally included in Analysis 1 (Fig. 1). There were no cases of hospital-acquired SARS-CoV-2 infection among the medical staff involved in the study, including the physicians, nurses, and physical therapists, during the study period.

### Baseline patient characteristics

The median duration of IMV was 9 days, and patients with duration of ventilation > 9 days were included in the prolonged IMV group. The baseline characteristics of the total patient population as well as subgroups stratified according to duration of ventilation are shown in Table 1. The overall study population consisted of patients with a median age of 60 years (85.9% male) and the clinical frailty scale score was low. The Krebs von den Lungen-6 (KL-6) level was significantly higher in the prolonged than short IMV group, but there were no significant differences between the groups in any other baseline characteristics, including age, sex, body mass index, severity score, clinical frailty scale score, or chronic medical conditions. Although the rates of extracorporeal membrane oxygenation and tracheostomy treatment were significantly higher in the prolonged than short IMV group, the rates of other ICU treatments, including neuromuscular blockade, were not significantly different between the two groups. The duration of ventilation was significantly longer in the prolonged than short IMV group (16 days [IQR 11–25 days] versus 7 days [IQR 5–8 days], respectively, *P* < 0.001).

**Table 1:** Baseline characteristics at ICU admission and ICU therapy (Analysis 1).

Factor	Overall (n = 64)	Short IMV (n = 34; 53%)	Prolonged IMV (n = 30; 47%)	*P* value
Age (yrs)	60 [52–66]	57 [51–66]	60 [54–68]	0.480
≥ 65 (%)	19 (29.7)	10 (29.4)	9 (30.0)	1.000
Male (%)	55 (85.9)	27 (79.4)	28 (93.3)	0.156
BMI (kg/m^2^)	25.5 [23.4–29.1]	25.9 [23.4–28.7]	25.2 [23.3–29.4]	0.861
< 25	29 (45.3)	15 (44.1)	14 (46.7)	0.806
25 to < 30	22 (34.4)	13 (38.2)	9 (30.0)	
≥ 30	13 (20.3)	6 (17.6)	7 (23.3)	
4C mortality score	11 [9–13]	11 [9–13]	12 [9–13]	0.432
SOFA score	10 [8–11]	10 [7–11]	10 [8–11]	0.501
APACHE II score	21 [19–24]	21 [20–25]	21 [18–24]	0.358
PaO_2_/FiO_2_ ratio	114 [76–157]	114 [79–150]	117 [75–202]	0.726
Clinical frailty scale score	3 [2–3]	3 [2–3]	3 [2–3]	0.316
Living alone (%)	21 (32.8)	9 (26.5)	12 (40.0)	0.294
Transfer from other hospitals (%)	53 (82.8)	31 (91.2)	22 (73.3)	0.096
Comorbidities (%)				
Hypertension	25 (39.1)	12 (35.3)	13 (43.3)	0.610
Diabetes mellitus	21 (32.8)	11 (32.4)	10 (33.3)	1.000
Heart disease	12 (18.8)	4 (11.8)	8 (26.7)	0.199
Cerebrovascular disease	7 (10.9)	2 (5.9)	5 (16.7)	0.238
Chronic pulmonary disease	2 (3.1)	1 (2.9)	1 (3.3)	1.000
Renal dysfunction	23 (35.9)	11 (32.4)	12 (40.0)	0.606
Cancer	10 (15.6)	4 (11.8)	6 (20.0)	0.495
Laboratory data				
Albumin (g/dL)	2.8 [2.6–3.0]	2.8 [2.6–2.9]	2.9 [2.5–3.2]	0.534
Hemoglobin (g/dL)	13.7 [12.7–14.9]	14.1 [13.0–15.0]	13.2 [12.3–14.7]	0.050
BUN (mg/dL)	23 [17–28]	24 [17–28]	22 [17–27]	0.957
Creatinine (mg/dL)	0.79 [0.67–1.12]	0.78 [0.64–1.06]	0.89 [0.68–1.20]	0.360
D-dimer (ug/mL)	1.50 [0.80–2.92]	1.30 [0.70–2.40]	1.90 [1.10–4.00]	0.297
CRP (mg/dL)	6.70 [3.92–11.37]	6.63 [4.25–11.48]	6.73 [3.02–9.85]	0.756
BNP (pg/mL)	26 [10–62]	27 [9–51]	24 [11–100]	0.561
KL-6 (U/mL)	392 [281–497]	349 [266–420]	472 [339–646]	0.013
ICU therapy (%)				
ECMO	7 (10.9)	0 (0.0)	7 (23.3)	0.003
Prone position	32 (50.0)	15 (44.1)	17 (56.7)	0.453
Tracheostomy	13 (20.3)	0 (0.0)	13 (43.3)	< 0.001
Steroid pulse therapy	47 (73.4)	24 (70.6)	23 (76.7)	0.777
Neuromuscular blockade	25 (39.1)	11 (32.4)	14 (46.7)	0.307
Duration of sedation (days)	7 [4–9]	5 [3–6]	9 [8–14]	< 0.001
Duration of IMV (days)	9 [6–15]	7 [5–8]	16 [11–25]	< 0.001

Values are expressed as n (%) or median [interquartile range].

*APACHE II* acute physiology and chronic health evaluation II, *BMI* body mass index, *BNP* B-type natriuretic peptide, *BUN* blood urea nitrogen, *CRP* C-reactive protein, *ECMO* extracorporeal membrane oxygenation, *ICU* intensive care unit, *KL-6* Krebs von den Lungen-6, *IMV* invasive mechanical ventilation, *PaO*_*2*_*/FiO*_*2*_ partial pressure of oxygen/fraction of inspired oxygen, *SOFA* sequential organ failure assessment.

### Assessments at ICU discharge (Analysis 1)

**Figure 2:**
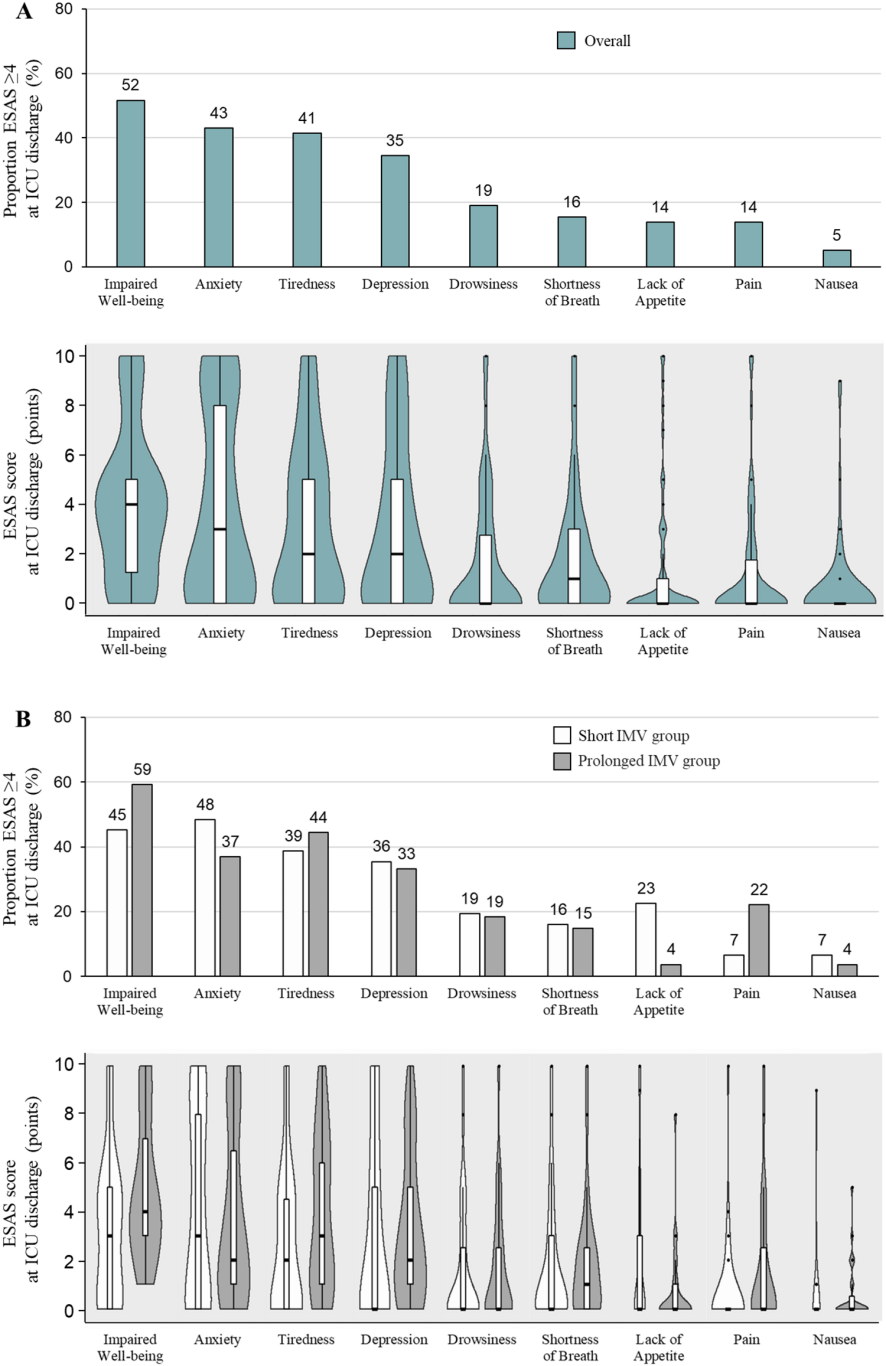
Proportion of patients with moderate-to-severe symptoms according to ESAS score at ICU discharge (Analysis 1). ESAS—edmonton symptom assessment system, ICU—intensive care unit, IMV—invasive mechanical ventilation.

The time to first mobilization and LOS in the ICU were significantly longer in the prolonged than short IMV group (Table 2). Both MRC sum score and handgrip strength at ICU discharge were significantly lower, and the percentages of patients with muscle weakness (MRC sum score < 48 points, and handgrip strength < 11 kg for males and < 7 kg for females) were significantly higher in the prolonged than short IMV group (46.7% vs. 11.8%, *P* = 0.002 and 43.3% vs. 14.7%, *P* = 0.011, respectively). ICU mobility scale, GRT, FTT, and ESAS sum scores at were not significantly different between the short and prolonged IMV groups. The symptoms were similar in both the short and prolonged IMV groups, and those most frequently reported as moderate-to-severe at ICU discharge were impaired well-being (52%), anxiety (43%), tiredness (41%), and depression (35%), but not shortness of breath (Fig. 2). There were no moderate-to-severe symptoms in 30% of the total study population. The prolonged IMV group had a significantly lower Barthel Index than the short IMV group (25 [IQR 5–85] versus 5 [IQR 0–45], respectively, *P* = 0.040) (Table 2).

**Table 2:** ICU outcomes, physical function, and symptom burden at ICU discharge (Analysis 1).

Factor	Overall (n = 64)	Short IMV (n = 34; 53%)	Prolonged IMV (n = 30; 47%)	*P* value
Time to first mobilize (days)	9 [7–13]	7 [5–8]	12 [11–20]	< 0.001
ICU LOS (days)	11 [8–18]	8 [6–10]	19 [13–31]	< 0.001
ICU Mobility Scale at ICU discharge	3 [1–4]	3 [1–5]	3 [1–3]	0.170
Physical function at ICU discharge				
MRC sum score (points)	56 [46–60]	60 [58–60]	51 [32–55]	< 0.001
< 48 points	18 (28.1)	4 (11.8)	14 (46.7)	0.002
Handgrip strength (kg)	15.2 [8.4–22.5]	18.0 [11.3–24.5]	10.6 [6.1–16.5]	0.003
Male < 11 kg, female < 7 kg	18 (28.1)	5 (14.7)	13 (43.3)	0.011
Male < 28 kg, female < 18 kg	54 (88.5)	26 (78.8)	28 (100.0)	0.013
Grip and release test score (/10 s)	17 [10–23]	17 [10–23]	16 [8–22]	0.481
Foot tapping test score (/10 s)	14 [9–20]	15 [11–18]	12 [0–21]	0.181
ESAS sum score at ICU discharge (points)	19 [8–33]	19 [5–32]	19 [9–33]	0.415
Post-ICU LOS (days)	4 [1–7]	4 [2–6]	4 [0–7]	0.532
Readmitted to ICU (%)	5 (7.8)	1 (2.9)	4 (13.3)	0.177
Discharged home (%)	15 (23.4)	11 (32.4)	26 (13.3)	0.085
Barthel Index at hospital discharge	15 [0–75]	25 [5–85]	5 [0–45]	0.040

*ESAS* edmonton symptom assessment system, *ICU* intensive care unit, *LOS* length of stay, *MRC* medical research council, *IMV* invasive mechanical ventilation.

Values are expressed as n (%) or median [interquartile range].

### Assessments at hospital discharge (Analysis 2)

**Figure 3:**
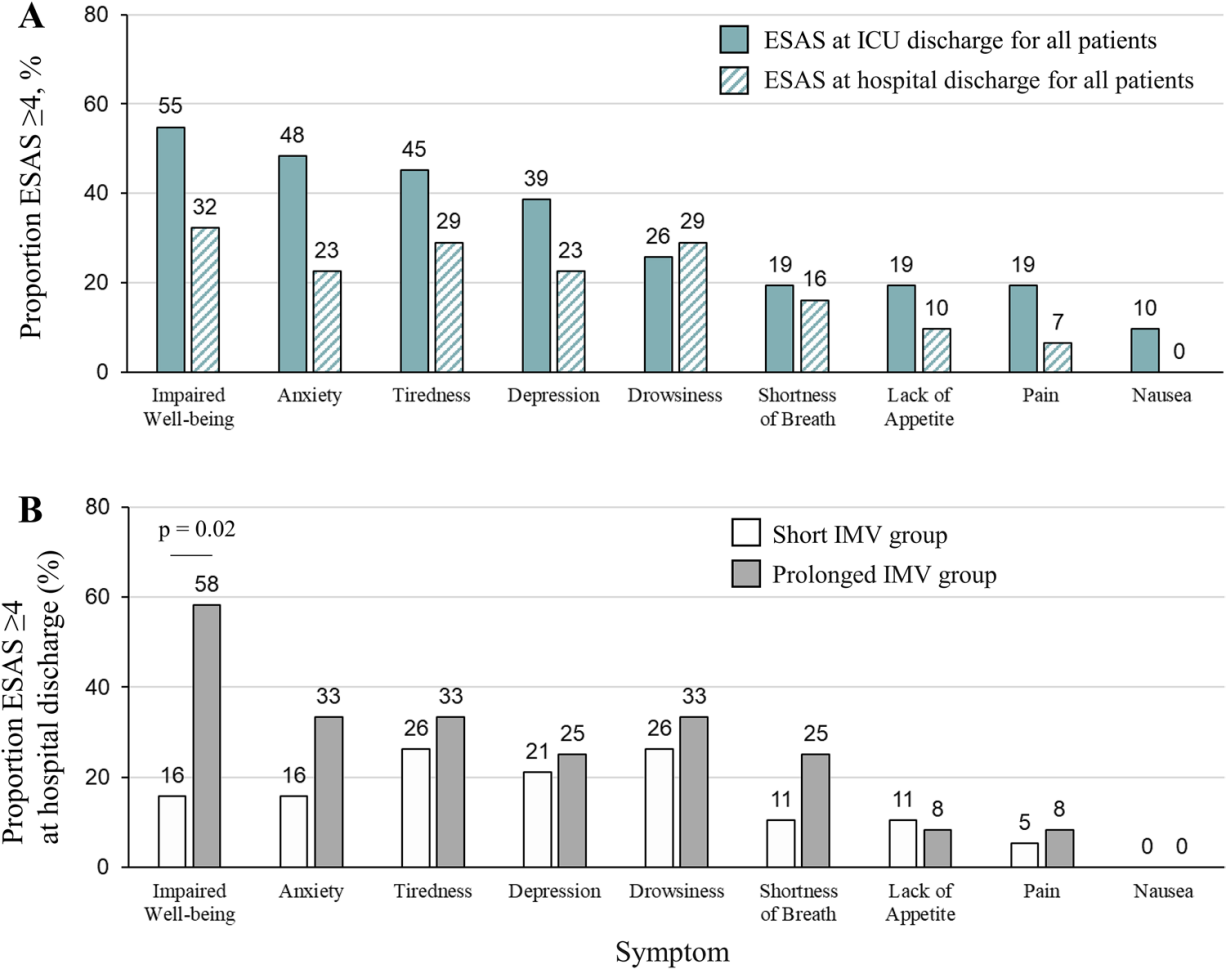
Proportion of patients with moderate-to-severe symptoms according to ESAS score after ICU discharge (Analysis 2). ESAS—edmonton symptom assessment system, ICU—intensive care unit, IMV—invasive mechanical ventilation.

Analysis 2 was performed in the population of 32 patients who received rehabilitation after ICU discharge and completed both ICU and hospital discharge assessments (Fig. 1). Both Analyses 1 and 2 showed similar associations between baseline characteristics and IMV duration (Table S1). The ICU mobility scale score increased significantly from ICU discharge to hospital discharge in both the short and prolonged IMV groups (Table 3). Although the ICU mobility scale score at hospital discharge was lower in the prolonged than short IMV group, the difference was not statistically significant (*P* = 0.070). Although both MRC sum score and handgrip strength increased significantly from ICU discharge to hospital discharge in the prolonged IMV group, MRC sum score at hospital discharge was significantly lower in the prolonged than short IMV group (*P* = 0.004). There were no significant differences between the short and prolonged IMV groups in the rates of low MRC sum score (< 48 points) (5.9% vs. 15.4%, respectively, *P* = 0.565) or low handgrip strength (< 28 kg for males and < 18 kg for females) (75.0% vs. 100%, respectively, *P* = 0.107). Although the short IMV group showed a significant decrease in ESAS sum score between ICU and hospital discharge, the decrease in the prolonged IMV group was not significant, and the ESAS sum score at hospital discharge was significantly higher in the prolonged than short IMV group (*P* = 0.024). The symptoms most frequently reported as moderate-to-severe at hospital discharge in the whole study population were impaired well-being (32%), anxiety (23%), tiredness (29%), depression (23%), and drowsiness (29%) (Fig. 3). The rates of symptoms were similar in both the short and prolonged IMV groups, with the exception of impaired well-being (16% vs. 58%, respectively, *P* = 0.020). There were no moderate-to-severe symptoms in 37% of patients in the total study population, and there was no significant difference in Barthel Index between the two groups in Analysis 2.

**Table 3:** Physical function and symptom burden after ICU discharge, and hospital outcomes (Analysis 2).

Factor	Overall (n = 32)	Short IMV (n = 19; 59%)	Prolonged IMV (n = 13; 41%)	*P* value
Post-ICU LOS (days)	6 [50–14]	5 [3–7]	7 [7–36]	0.002
Readmitted to ICU (%)	3 (9.4)	0 (0.0)	3 (23.1)	0.058
ICU mobility scale				
At ICU discharge	3 [1–4]	3 [1–4]	3 [3–3]	0.966
At hospital discharge	8 [6–10]*	9 [6–10]*	6 [4–9]*	0.070
MRC sum score (points)				
At ICU discharge	58 [46–60]	60 [57–60]	46 [29–56]	0.006
At hospital discharge	60 [56–60]*	60 [60–60]	56 [53–60]*	0.004
Handgrip strength (kg)				
At ICU discharge	14.7 [8.5–23.1]	17.5 [10.2–24.7]	11.3 [5.9–15.2]	0.060
At hospital discharge	16.2 [11.5–23.5]*	18.7 [13.3–27.6]	15.8 [8.4–19.7]*	0.125
Grip and release (/10 s)				
At ICU discharge	14 [10–21]	14 [10–24]	16 [9–21]	0.690
At hospital discharge	22 [16–28]*	22 [15–28]*	22 [18–23]	1.000
Foot tapping (/10 s)				
At ICU discharge	14 [10–18]	14 [11–16]	14 [7–23]	1.000
At hospital discharge	20 [15–25]*	19 [14–24]*	20 [16–27]	0.589
ESAS sum score (points)				
At ICU discharge	20 [9–35]	20 [4–34]	30 [16–38]	0.109
At hospital discharge	13 [3–21]*	4 [1–18]*	17 [13–25]	0.024
Discharged home (%)	21 (34.4)	11 (42.1)	10 (23.1)	0.450
Barthel Index at hospital discharge	55 [10–85]	75 [15–90]	50 [10–60]	0.453

*ESAS* edmonton symptom assessment system, *ICU* intensive care unit, *LOS* length of stay, *MRC* medical research council, *IMV* invasive mechanical ventilation.

Values are expressed as n (%) or median [interquartile range].

*Within-group comparison, *P* < 0.05.

### Assessments at 6 months after ICU discharge (Analysis 3)

Analysis 3 at 6 months after ICU discharge was performed in a total of 42 patients (Fig. 1). EQ-VAS and EQ-5D-5L summary index were significantly lower (80 [IQR 70–90] vs. 90 [IQR 80–95], *P* = 0.046 and 0.82 [IQR 0.62–1.00] vs. 0.89 [IQR 0.89–1.00], *P* = 0.023, respectively), and all domains, with the exception of pain/discomfort, in the EQ-5D-5L were significantly worse in the prolonged than short IMV group (Table 4 and Table S2).

**Table 4:** Quality of life outcomes at 6 months (Analysis 3).

Factor	Overall (n = 42)	Short IMV (n = 21; 50%)	Prolonged IMV (n = 21; 50%)	*P* value
EQ-VAS	85 [70–90]	90 [80–95]	80 [70–90]	0.046
EQ-5D-5L summary index	0.89 [0.73–1.00]	0.89 [0.89–1.00]	0.82 [0.62–1.00]	0.023
Mobility	1 [1–2]	1 [1–1]	2 [1–2]	0.003
Self-care	1 [1–1]	1 [1–1]	1 [1–2]	0.009
Usual activities	1 [1–2]	1 [1–1]	2 [1–2]	0.006
Pain/discomfort	1 [1–2]	1 [1–2]	1 [1–2]	0.159
Anxiety/depression	1 [1–2]	1 [1–1]	1 [1–2]	0.041

*EQ-VAS* EuroQol visual analogue scale, *EQ-5D-5L* EuroQol five-dimension five-level, *IMV* invasive mechanical ventilation.

Values are expressed as n (%) or median [interquartile range].

## Discussion

This study was performed to investigate the physical function and mental health trajectories after ICU discharge in patients with severe COVID-19 requiring IMV. There were no significant differences in baseline characteristics, including age, frailty status, and chronic medical conditions at ICU admission, between the short and prolonged IMV groups in the present study. Prolonged IMV was significantly associated with longer time to first mobilization and LOS in the ICU and reduced muscle strength at ICU discharge, although ICU mobility scale, MRC sum score, and handgrip strength showed significant improvements in these patients between the time of ICU discharge and hospital discharge. The most commonly reported moderate-to-severe symptoms after ICU were impaired well-being, anxiety, tiredness, and depression, but not shortness of breath, in patients with severe COVID-19. Symptom burden assessed according to the ESAS sum score showed no significant improvement after ICU discharge in the prolonged IMV group. The prolonged IMV group showed significantly poorer QOL dimensions, i.e., mobility, self-care, usual activities, and anxiety/depression, as well as QOL score in comparison to the short IMV group at 6 months after ICU discharge.

Although COVID-19 patients with respiratory failure frequently require prolonged IMV support, the in-hospital mortality rate has decreased from 30% at the start of the pandemic to below 20% at present^25^. Consistent with large-scale registries of severe COVID-19^26,27^, the median IMV duration was 9 (IQR 6–15) days in the present study, which was markedly longer than in other critically ill patients in the ICU^28^. Previous studies showed that prolonged IMV in patients with acute respiratory failure has detrimental effects on both physical function and mental health^4,5^, with a number of sequelae including generalized weakness seen on long-term follow-up, which has been termed as post-intensive care syndrome^29^. ICU-acquired weakness increases both in-hospital and long-term mortality risks, duration of hospitalization, healthcare-related costs, and likelihood of prolonged care in rehabilitation centers, and is associated with long-term reduction of QOL^30^. The reported rates of ICU-acquired weakness have a wide range from 9 to 86%, which may have been due to differences in both the definition of the condition and in patient characteristics between studies^31^. Higher disease severity and greater numbers of both comorbidities and of organs with dysfunction are associated with increased risk of ICU-acquired weakness^16^. There were no significant differences in baseline characteristics, including age, frailty status, and chronic medical conditions, at ICU admission between the short and prolonged IMV groups in the present study. However, 28.1% of patients in our total study population had MRC sum score < 48 points and 46.7% of those in the prolonged IMV group had muscle weakness, which were similar to the results of previous studies showing that prolonged IMV was associated with an increased incidence of ICU-acquired weakness^32,33^. Persistent inflammation in patients with multiple organ dysfunction after acute proinflammatory-driven critical illness was suggested to be strongly correlated with end-organ muscle inflammation, acute muscle wasting, and poor long-term functional outcome^16^. In addition, patients requiring prolonged IMV have been shown to have longer LOS in the ICU and require deep sedation, neuromuscular blockade, and/or placement in the prone position, all of which are significant risk factors for ICU-acquired weakness^6^, and these factors may have been responsible for the occurrence of ICU-acquired weakness in patients with severe COVID-19 in the present study. Several studies showed that 20–30% of surviving COVID-19 patients in cohorts with mixed severity of illness had symptoms of dyspnea, anxiety, depression, fatigue, and weakness even at 6 months after discharge, and patients with greater severity of illness tended to show more symptoms^10,34,35^. Regardless of the duration of IMV, more than 33% of patients with severe COVID-19 in the present study reported moderate-to-severe impaired well-being, anxiety, fatigue, and depression at ICU discharge. A recent study also reported that ICU-treated COVID-19 patients showed more severe long-term cognitive impairment in comparison to patients with less severe acute COVID-19 or non-COVID controls^36^. Taken together, these observations indicate a need for further studies of the burden of disability across all areas of post-intensive care syndrome.

The results presented here have important implications both for clinical practice and for the design of future clinical studies of severe COVID-19. Although early rehabilitation for COVID-19 in the ICU has been reported to be both safe and feasible^7,37,38^, early rehabilitation may be delayed in the ICU for a number of reasons, all of which are exacerbated by staffing shortages and infection control, overwhelmed hospital capacity, and the obesity status and severity of respiratory failure of patients. In the present study, prolonged IMV was significantly associated with longer time to first mobilization and LOS in the ICU, suggesting that new interventions, such as electrical muscle stimulation therapy, should be implemented in the ICU to prevent functional decline in patients with severe COVID-19^39^. Mobility status and physical function were both reported to be improved by supervised rehabilitation therapy in COVID-19 patients in the post-acute setting regardless of the severity of disease^40–42^. Continued rehabilitation after ICU discharge was shown to result in significant improvement in mobility at hospital discharge in patients with severe COVID-19 regardless of the duration of IMV in the present study. Muscle strength was also significantly increased after ICU discharge in the prolonged IMV group, while the MRC sum score remained significantly lower than in the short IMV group until hospital discharge, and all patients in the prolonged IMV group met the frailty criteria of low handgrip strength at hospital discharge. The prolonged IMV group also showed no significant improvement of symptom burden after ICU discharge, and even at 6 months after ICU discharge, mobility, self-care, usual activities, and anxiety/depression and health-related QOL were significantly worse in the prolonged IMV group than the short IMV group. The impairments in both physical and mental health in COVID-19 patients after discharge were reported to be closely correlated with one another^10^. Although the minimal clinically important differences in severe COVID-19 patients for the assessments used in this study are unknown and caution is required in interpreting the results of this study, these observations indicate the need for a coordinated multidisciplinary approach to support survival and ICU recovery of patients with severe COVID-19.

This study had several limitations. First, this was a single-center observational study in a small population with only limited follow-up. Second, the study population consisted only of Asian COVID-19 patients, and the findings may therefore not be generalizable to other populations. Third, this study included only COVID-19 patients requiring IMV, and these patients were very heavily sedated and curarized for long periods. Therefore, these impairments were not related to SARS-CoV-2, but may have been related to treatment in the ICU. Fourth, the analysis did not include patients who died, and the worst clinical condition of patients should also be considered in interpretation of the results. Finally, the lack of assessment of muscle strength and symptom burden after hospital discharge and health-related QOL during hospitalization prevented us from reaching definitive conclusions about the overall long-term recovery of physical function and mental health in these patients.

## Conclusions

The results presented here showed that most surviving COVID-19 patients requiring IMV had not fully recovered after ICU discharge and still had substantial impairments in both physical function and mental health. Prolonged periods of IMV showed negative effects not only immediately but also 6 months after ICU discharge. This study suggested the need for the implementation of a proactive multidisciplinary approach from early in the ICU to long-term follow-up after hospital discharge in surviving COVID-19 patients who required ventilation.

### Supplementary Information

Supplementary Information.

## Data Availability

The datasets used and/or analyzed during the current study are available from the corresponding author on reasonable request.

## Abbreviations

COVID-19: Coronavirus disease 2019
SARS-CoV-2: Severe acute respiratory syndrome coronavirus 2
IMV: Invasive mechanical ventilation
ICU: Intensive care unit
QOL: Quality of life
LOS: Length of stay
4C: Coronavirus clinical characterisation consortium
APACHE II: Acute physiology and chronic health evaluation II
SOFA: Sequential organ failure assessment
MRC: Medical research council
GRT: Grip and release test
FTT: Foot tapping test
ESAS: Edmonton symptom assessment system
ADL: Activities of daily living
EQ-5D-5L: EuroQol five-dimension five-level
EQ-VAS: EuroQol visual analogue scale
IQR: Interquartile range
KL-6: Krebs von den Lungen-6
