# Supplementary material for: Physical function and mental health trajectories in COVID-19 patients following invasive mechanical ventilation: a prospective observational study
Source: Sci Rep. 2023 Sep 4;13:14529. doi: 10.1038/s41598-023-41684-3 (PMC10477337; doi:10.1038/s41598-023-41684-3)
Supplement: Supplementary file 1 — Supplementary Information. [file 41598_2023_41684_MOESM1_ESM.pdf]

Physical function and mental health trajectories in COVID-19 patients following invasive mechanical ventilation: a prospective observational study

Hiromasa Yamamoto[1†], Shinya Tanaka[1†], Daisuke Kasugai[2*], Miho Shimizu[3], Yohei Tsuchikawa[1], Yuto Hori[1], Yuki Fugane[1], Takayuki Inoue[1], Motoki Nagaya[1], Norihito Omote[4], Michiko Higashi[2], Takanori Yamamoto[2], Naruhiro Jingushi[2], Atsushi Numaguchi[2], Yukari Goto[2], Yoshihiro Nishida[1,5]

[1] Department of Rehabilitation, Nagoya University Hospital, Nagoya, Japan

[2] Department of Emergency and Critical Care Medicine, Nagoya University Graduate School of Medicine, Nagoya, Japan

[3] Department of Rehabilitation, Mie University Hospital, Tsu, Japan

[4] Department of Respiratory Medicine, Nagoya University Graduate School of Medicine, Nagoya, Japan

[5] Department of Orthopaedic Surgery, Nagoya University Graduate School of Medicine, Nagoya, Japan

[†]Hiromasa Yamamoto and Shinya Tanaka have contributed equally to this work.

Table S1. Baseline Characteristics, ICU therapy, and ICU outcomes (Analysis 2)

Factor	Overall (n=32)	Short IMV (n=19; 59%)	Prolonged IMV (n=13; 41%)	*P* value
Age (yrs)	60 [52-69]	56 [51-69]	63 [57-68]	0.198
≥65 (%)	10 (31.2)	6 (31.6)	4 (30.8)	1.000
Male (%)	55 (85.9)	27 (79.4)	28 (93.3)	0.156
BMI (kg/m^2)	24.7 [22.7-26.6]	25.3 [22.6-27.6]	23.8 [23.2-25.3]	0.432
<25	18 (56.2)	9 (47.4)	9 (69.2)	0.515
25 to <30	10 (31.2)	7 (36.8)	3 (23.1)	
≥30	4 (12.5)	3 (15.8)	1 (7.7)	
4C mortality score	12 [10-13]	10 [10-13]	12 [11-14]	0.439
SOFA score	10 [8-11]	10 [8-12]	10 [9-11]	0.727
APACHE II score	21 [19-24]	21 [19-24]	21 [18-25]	0.923
PaO2/FiO2 ratio	121 [75-157]	115 [77-144]	148 [75-209]	0.409
Clinical frailty scale score	3 [2-3]	3 [2-3]	3 [2-3]	0.623
Living alone (%)	10 (31.2)	4 (21.1)	6 (46.2)	0.244
Transfer from other hospitals (%)	24 (75.0)	17 (89.5)	7 (53.8)	0.038
Comorbidities (%)				
Hypertension	15 (46.9)	8 (42.1)	7 (53.8)	0.720
Diabetes mellitus	11 (34.4)	6 (31.6)	5 (38.5)	0.721
Heart disease	5 (15.6)	2 (10.5)	3 (23.1)	0.374
Cerebrovascular disease	2 (6.2)	0 (0.0)	2 (15.4)	0.157
Chronic pulmonary disease	1 (3.1)	1 (5.3)	0 (0.0)	1.000
Renal dysfunction	16 (50.0)	8 (42.1)	8 (61.5)	0.473
Cancer	6 (18.8)	3 (15.8)	3 (23.1)	0.666
Laboratory data				
Albumin (g/dL)	2.9 [2.7-3.1]	2.8 [2.7-3.1]	3.0 [2.7-3.4]	0.375
Hemoglobin (g/dL)	13.7 [12.6-15.2]	14.7 [13.3-15.4]	12.7 [10.8-13.6]	0.068
BUN (mg/dL)	23 [18-29]	26 [17-29]	20 [19-27]	0.715
Creatinine (mg/dL)	0.89 [0.72-1.21]	0.79 [0.69-1.21]	0.97 [0.84-1.21]	0.318
D-dimer (ug/mL)	1.45 [0.65-2.42]	1.25 [0.55-1.80]	2.40 [1.70-4.60]	0.111
CRP (mg/dL)	6.56 [4.86-11.08]	6.53 [4.86-11.09]	8.84 [4.26-10.11]	1.000
BNP (pg/mL)	22 [11-87]	20 [8-44]	53 [12-110]	0.414
KL-6 (U/mL)	370 [251-474]	370 [266-458]	370 [205-489]	0.632
ICU therapy (%)				
ECMO	1 (3.1)	0 (0.0)	1 (7.7)	0.406
Prone position	16 (50.0)	9 (47.4)	7 (53.8)	1.000
Tracheostomy	4 (12.5)	0 (0.0)	4 (30.8)	0.020
Steroid pulse therapy	27 (84.4)	17 (89.5)	10 (76.9)	0.374
Neuromuscular blockade	8 (25.0)	5 (26.3)	3 (23.1)	1.000
Duration of sedation (days)	7 [4-8]	5 [3-6]	9 [8-11]	<0.001
Duration of ventilation (days)	9 [7-11]	7 [5-9]	15 [10-21]	<0.001
ICU outcomes				
Time to first rehabilitation (days)	6 [4-7]	5 [4-7]	7 [5-13]	0.047
Time to first mobilize (days)	8 [7-12]	7 [5-8]	12 [10-17]	0.002
ICU LOS (days)	11 [9-18]	10 [7-11]	18 [13-25]	<0.001

Values are expressed as n (%) or median [interquartile range].

APACHE II, Acute Physiology and Chronic Health Evaluation II; BMI, body mass index; BNP, B-type natriuretic peptide; BUN, blood urea nitrogen; CRP, C-reactive protein; ECMO, extracorporeal membrane oxygenation; ICU, intensive care unit; KL-6, Krebs von den Lungen-6; IMV, invasive mechanical ventilation; LOS, length of stay; PaO_2/FiO_2, partial pressure of oxygen/fraction of inspired oxygen; SOFA, Sequential Organ Failure Assessment.

Table S2. EQ-5D-5L score in each dimension at 6 months (Analysis 3)

Factor	Overall (n=42)	Short IMV (n=21; 50%)	Prolonged IMV (n=21; 50%)
Mobility			
Score 1: I have no problems with walking around	27 (64.3)	18 (85.7)	9 (42.9)
Score 2: I have slight problems with walking around	10 (23.8)	3 (14.3)	7 (33.3)
Score 3: I have moderate problems with walking around	3 (7.1)	0 (0)	3 (14.3)
Score 4: I have severe problems with walking around	2 (4.8)	0 (0)	2 (9.5)
Score 5: I am unable to walk around	0 (0)	0 (0)	0 (0)
Self-care			
Score 1: I have no problems with washing or dressing myself	36 (85.7)	21 (100.0)	15 (71.4)
Score 2: I have slight problems with washing or dressing myself	6 (14.3)	0 (0)	6 (28.6)
Score 3: I have moderate problems with washing or dressing myself	0 (0)	0 (0)	0 (0)
Score 4: I have severe problems with washing or dressing myself	0 (0)	0 (0)	0 (0)
Score 5: I am unable to wash or dress myself	0 (0)	0 (0)	0 (0)
Usual activities			
Score 1: I have no problems doing my usual activities	28 (66.7)	18 (85.7)	10 (47.6)
Score 2: I have slight problems doing my usual activities	10 (23.8)	3 (14.3)	7 (33.3)
Score 3: I have moderate problems doing my usual activities	3 (7.1)	0 (0)	3 (14.3)
Score 4: I have severe problems doing my usual activities	0 (0)	0 (0)	0 (0)
Score 5: I am unable to do my usual activities	1 (2.4)	0 (0)	1 (4.8)
Pain/discomfort			
Score 1: I have no pain or discomfort	25 (59.5)	14 (66.7)	11 (52.4)
Score 2: I have slight pain or discomfort	12 (28.6)	7 (33.3)	5 (23.8)
Score 3: I have moderate pain or discomfort	5 (11.9)	0 (0)	5 (23.8)
Score 4: I have severe pain or discomfort	0 (0)	0 (0)	0 (0)
Score 5: I have extreme pain or discomfort	0 (0)	0 (0)	0 (0)
Anxiety/depression			
Score 1: I am not anxious or depressed	28 (66.7)	17 (81.0)	11 (52.4)
Score 2: I am slightly anxious or depressed	12 (28.6)	4 (19.0)	8 (38.1)
Score 3: I am moderately anxious or depressed	1 (2.4)	0 (0)	1 (4.8)
Score 4: I am severely anxious or depressed	1 (2.4)	0 (0)	1 (4.8)
Score 5: I am extremely anxious or depressed	0 (0)	0 (0)	0 (0)

Values are expressed as n (%).

EQ-5D-5L, EuroQol five-dimension five-level; IMV, invasive mechanical ventilation.
